# Deep Convolutional Neural Network for Ulcer Recognition in Wireless Capsule Endoscopy: Experimental Feasibility and Optimization

**DOI:** 10.1155/2019/7546215

**Published:** 2019-09-18

**Authors:** Sen Wang, Yuxiang Xing, Li Zhang, Hewei Gao, Hao Zhang

**Affiliations:** ^1^Key Laboratory of Particle & Radiation Imaging (Tsinghua University), Ministry of Education, Beijing, China; ^2^Department of Engineering Physics, Tsinghua University, Beijing 100084, China; ^3^Ankon Technologies Co., Ltd., Wuhan, Shanghai, China

## Abstract

Wireless capsule endoscopy (WCE) has developed rapidly over the last several years and now enables physicians to examine the gastrointestinal tract without surgical operation. However, a large number of images must be analyzed to obtain a diagnosis. Deep convolutional neural networks (CNNs) have demonstrated impressive performance in different computer vision tasks. Thus, in this work, we aim to explore the feasibility of deep learning for ulcer recognition and optimize a CNN-based ulcer recognition architecture for WCE images. By analyzing the ulcer recognition task and characteristics of classic deep learning networks, we propose a HAnet architecture that uses ResNet-34 as the base network and fuses hyper features from the shallow layer with deep features in deeper layers to provide final diagnostic decisions. 1,416 independent WCE videos are collected for this study. The overall test accuracy of our HAnet is 92.05%, and its sensitivity and specificity are 91.64% and 92.42%, respectively. According to our comparisons of F1, F2, and ROC-AUC, the proposed method performs better than several off-the-shelf CNN models, including VGG, DenseNet, and Inception-ResNet-v2, and classical machine learning methods with handcrafted features for WCE image classification. Overall, this study demonstrates that recognizing ulcers in WCE images via the deep CNN method is feasible and could help reduce the tedious image reading work of physicians. Moreover, our HAnet architecture tailored for this problem gives a fine choice for the design of network structure.

## 1. Introduction

Gastrointestinal (GI) diseases pose great threats to human health. Gastric cancer, for example, ranks fourth among the most common type of cancers globally and is the second most common cause of death from cancer worldwide [1]. Conventional gastroscopy can provide accurate localization of lesions and is one of the most popular diagnostic modalities for gastric diseases. However, conventional gastroscopy is painful and invasive and cannot effectively detect lesions in the small intestine.

The emergence of wireless capsule endoscopy (WCE) has revolutionized the task of imaging GI issues; this technology offers a noninvasive alternative to the conventional method and allows exploration of the GI tract with direct visualization. WCE has been proven to have great value in evaluating focal lesions, such as those related to GI bleeding and ulcers, in the digestive tract [2].

**Figure 1:**
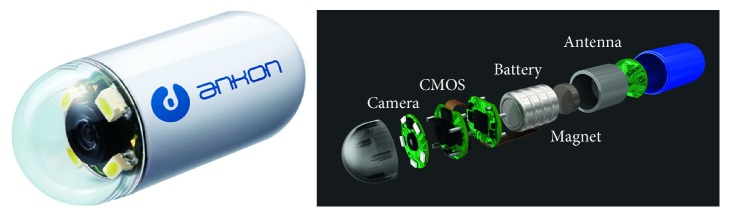
Illustration of a wireless capsule. This capsule is a product of Ankon Technologies Co., Ltd. (Wuhan, Shanghai, China).

WCE was first induced in 2000 by Given Imaging and approved for use by the U.S. Food and Drug Administration in 2001 [3]. In the examination phase, a capsule is swallowed by a patient and propelled by peristalsis or magnetic fields to travel along the GI tract [3, 4]. While travelling, the WCE takes colored pictures of the GI tract for hours at a frame rate of 2–4 photographs per second [3] and transmits the same to a data-recording device. The recorded images are viewed by physicians to arrive at a diagnosis.  Figure 1 illustrates a wireless capsule.

Examination of WCE images is a time-consuming and tedious endeavor for doctors because a single scan for a patient may include up to tens of thousands of images of the GI tract. Experienced physicians may spend hours reviewing each case. Furthermore, abnormal frames may occupy only a tiny portion of all of the images obtained [5]. Thus, physicians may miss the actual issue due to fatigue or oversight.

Several features have motivated researchers to turn to computer-aided systems, including improved ulcer detection, polyp recognition, and bleeding area segmentation [3, 5–15], to reduce the burden on physicians and guarantee diagnostic precision.

**Figure 2:**
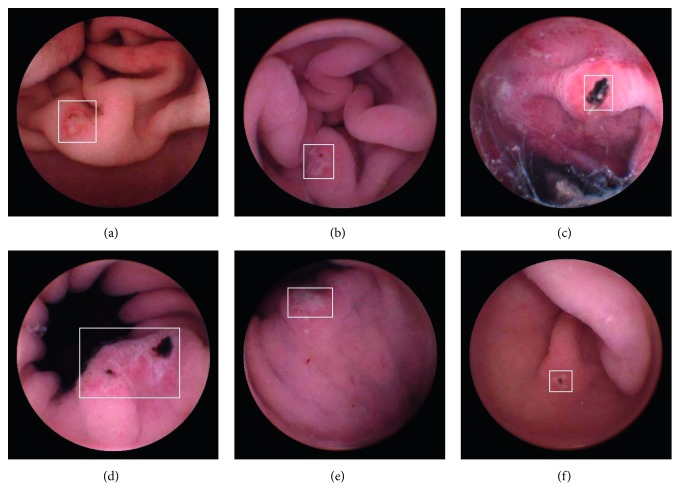
Typical WCE images of an ulcer. Ulcerated areas in each image are marked by a white box.

Ulcers are one of the most common lesions in the GI tract; an estimated 1 out of every 10 persons is believed to suffer from ulcers [13]. An ulcer is defined as an area of tissues destroyed by gastric juice and showing a discontinuity or break in a bodily membrane [9, 11]. The color and texture of the ulcerated area are different from those of a normal GI tract. Some representative ulcer frames in WCE videos are demonstrated in Figure 2. Ulcer recognition requires classification of each image in a WCE video as ulcerated or not, similar to the classification work in computer vision tasks.

Deep learning methods based on the convolutional neural network (CNN) have seen several breakthroughs in classification tasks in recent years. Considering the difficulty in mathematically describing the great variation in the shapes and features of abnormal regions in WCE images and the fact that deep learning is powerful in extracting information from data, we propose the application of deep learning methods to ulcer recognition using a large WCE dataset of big volume to provide adequate diversity. In this paper, we carefully analyze the problem of ulcer frame classification and propose a deep learning framework based on a multiscale feature concatenated CNN, hereinafter referred to as HAnet, to assist in the WCE video examination task of physicians. Our network is verified to be effective on a large dataset containing WCE videos of 1,416 patients.

Our main contributions can be summarized in terms of the following three aspects: (1) The proposed architecture adopts state-of-the-art CNN models to efficiently extract features for ulcer recognition. It incorporates a special design that fuses hyper features from shallow layers and deep features from deep layers to improve the recognition of ulcers at vastly distributed scales. (2) To the best of our knowledge, this work is the first experimental study to include a large dataset consisting of over 1,400 WCE videos from ulcer patients to explore the feasibility of deep CNN for ulcer diagnosis. Some representative datasets presented in published works are listed in Table 1. The 92.05% accuracy and 0.9726 ROC-AUC of our proposed model demonstrate its great potential for practical clinic applications. (3) An extensive comparison with different state-of-the-art CNN network structures is provided to evaluate the most promising network for ulcer recognition.

## 2. Materials and Methods

### 2.1. Abnormality Recognition in WCE Videos

Prior related methods for abnormality recognition in WCE videos can be roughly divided into two classes: conventional machine learning techniques with handcrafted features and deep learning methods.

Conventional machine learning techniques are usually based on manually selected handcrafted features followed by application of some classifier. Features commonly employed in conventional techniques include color and textural features.

Lesion areas are usually of a different color from the surrounding normal areas; for example, bleeding areas may present as red and ulcerated areas may present as yellow or white. Fu et al. [12] proposed a rapid bleeding detection method that extracts color feature in the RGB color space. Besides the RGB color space, other color spaces, like the HSI/HSV [9] and YCbCr [3], are also commonly used to extract features.

Texture is another type of feature commonly used for pattern recognition. Texture features include local binary patterns (LBP) and filter-based features [7]. An LBP descriptor is based on a simple binary coding scheme that compares each pixel with its neighbors [19]. The LBP descriptor, as well as its extended versions, such as uniform LBP [8] and monogenic LBP [20], has been adopted in various WCE recognition tasks. Filter-based features, such as Gabor filters and wavelet transforms, are widely used in WCE image recognition tasks for their ability to describe images in multistage space. In addition, different textural features can be combined for better recognition performance. As demonstrated in [8], the combination of wavelet transformation and uniform LBP can achieve automatic polyp detection with good accuracy.

CNN-based deep learning methods are known to show impressive performance. The error rate in computer vision challenges (e.g., ImageNet, COCO) has decreased rapidly with the emergence of various deep CNN architectures, such as AlexNet, VGGNet, GoogLeNet, and ResNet [21–28].

Many researchers have realized that handcrafted features merely encode partial information in WCE images [29] and that deep learning methods are capable of extracting powerful feature representations that can be used in WCE lesion recognition and depth estimation [6, 7, 15, 18, 30–34].

A framework for hookworm detection was proposed in [14]; this framework consists of an edge extraction network and a hookworm classification network. Inception modules are used to capture multiscale features to capture spatial correlations. The robustness and effectiveness of this method were verified in a dataset containing 440,000 WCE images of 11 patients. Yuan and Meng [7] proposed an autoencoder-based neural network model that introduces an image manifold constraint to a traditional sparse autoencoder to recognize polyps in WCE images. Manifold constraint can effectively enforce images within the same category to share similar features and keep images in different categories far way, i.e., it can preserve large intervariances and small intravariance among images. The proposed method was evaluated using 3,000 normal WCE images and 1,000 WCE images with polyps extracted from 35 patient videos. Utilizing temporal information of WCE videos with 3D convolution has also been explored for poly detection [33]. Deep learning methods are also adopted for ulcer diagnosis. There are also some investigations of ulcer recognition with deep learning methods [18, 35]. Off-the-shelf CNN models are trained and evaluated in these studies. Experimental results and comparisons in these studies clearly demonstrate the superiority of deep learning methods over conventional machine learning techniques.

From ulcer size analysis of our dataset, we find that most of the ulcers occupy only a tiny area in the whole image. Deep CNNs can inherently compute feature hierarchies layer by layer. Hyper features from shallow layers have high resolution but lack representation capacity; by contrast, deep features from deep layers are semantically strong but have poor resolution [36–38]. These features motivate us to propose a framework that fuses hyper and deep features to achieve ulcer recognition at vastly different scales. We will give detailed description of ulcer size analysis and the proposed method in Sections 2.2 and 2.3.

### 2.2. Ulcer Dataset

Our dataset is collected using a WCE system provided by Ankon Technologies Co., Ltd. (Wuhan, Shanghai, China). The WCE system consists of an endoscopic capsule, a guidance magnet robot, a data recorder, and a computer workstation with software for real-time viewing and controlling. The capsule is 28 mm × 12 mm in size and contains a permanent magnet in its dome. Images are recorded and transferred at a speed of 2 frames/s. The resolution of the WCE image is 480 × 480 pixels.

**Figure 3:**
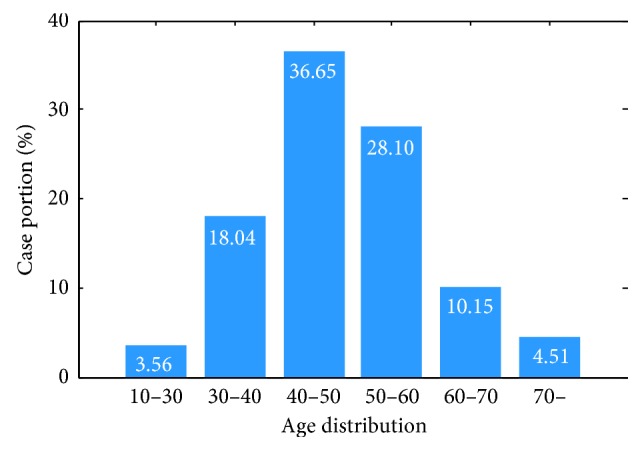
Age distribution of patients providing videos for this study. The horizontal axis denotes the age range and the vertical axis denotes the case portion.

The dataset used in this work to evaluate the performance of the proposed framework contains 1,416 WCE videos from 1,416 patients (males 73%, female 27%), i.e., one video per patient. The WCE videos are collected from more than 30 hospitals and 100 medical examination centers through the Ankon WCE system. Each video is independently annotated by at least two gastroenterologists. If the difference between annotation bounding boxes of the same ulcer is larger than 10%, an expert gastroenterologist will review the annotation and provide a final decision. The age distribution of patients is illustrated in Figure 3. The entire dataset consists of 1,157 ulcer videos and 259 normal videos. In total, 24,839 frames are annotated as ulcers by gastroenterologists. To balance the volume of each class, 24,225 normal frames are randomly extracted from normal videos for this study to match the 24,839 representative ulcer frames. A mask of diameter 420 pixels was used to crop the center area of each image in preprocessing. This preprocessing did not change the image size.

**Figure 4:**
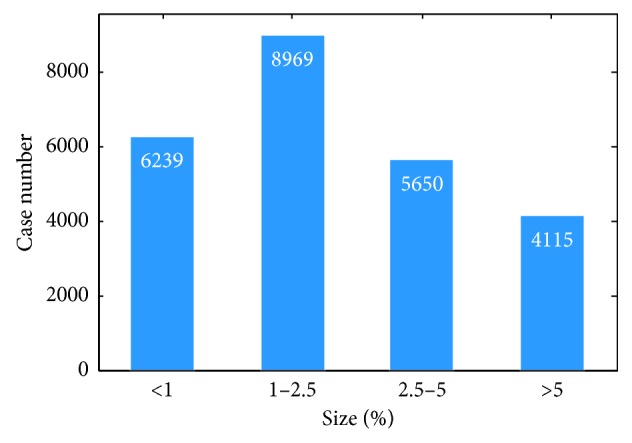
Distribution of ulcer size among patients. The horizontal axis denotes the ratio of ulcer area to the whole image, while the vertical axis presents the number of frames.

We plot the distribution of ulcer size in our dataset in Figure 4. The vertical and horizontal axes denote the number of images and the ratio of the ulcerated area to the whole image size, respectively. Despite the inspiring success of CNNs in ImageNet competition, ulcer recognition presents some challenge to the ImageNet classification task because lesions normally occupy only a small area of WCE images and the structures of lesions are rather subtle. In Figure 4, about 25% of the ulcers occupy less than 1% of the area of the whole image and more than 80% of the ulcers found occupy less than 5% of the area of the image. Hence, a specific design of a suitable network is proposed to account for the small ulcer problem and achieve good sensitivity.

### 2.3. HAnet-Based Ulcer Recognition Network with Fused Hyper and Deep Features

In this section, we introduce our design and the proposed architecture of our ulcer recognition network.

**Figure 5:**
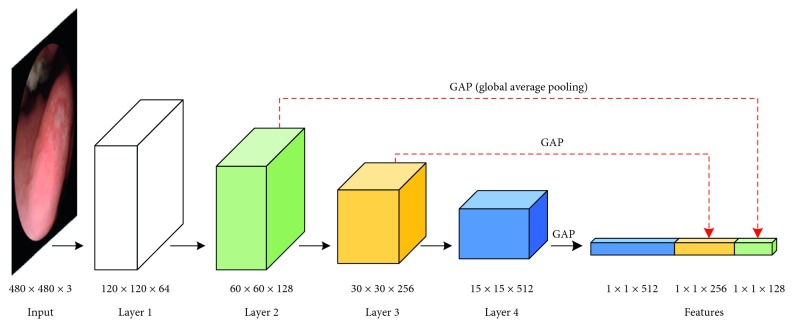
Ulcer recognition network framework. ResNet-34, which has 34 layers, is selected as the feature extractor. Here, we only display the structural framework for clarity. Detailed layer information can be found in Appendix.

Inspired by the design concept of previous works that deal with object recognition in vastly distributed scales [36–38], we propose an ulcer recognition network with a hyperconnection architecture (HAnet). The overall pipeline of this network is illustrated in Figure 5. Fundamentally, HAnet fuses hyper and deep features. Here, we use ResNet-34 as the base feature-extraction network because, according to our experiments (demonstrated in Section 3), it provides the best results. Global average pooling (GAP) [39] is used to generate features for each layer. GAP takes an average of each feature layer, so that it reduces tensors with dimensions *h* × *w* × *d* to 1 × 1 × *d*. Hyper features can be extracted from multiple intermediate layers (layers 2 and 3 in this case) of the base network; they are concatenated with the features of last feature-extraction layer (layer 4 in this case) to make the final decision.

Our WCE system outputs color images with a resolution of 480 × 480 pixels. Experiments by the computer vision community [36, 40] have shown that high-resolution input images are helpful to the performance of CNN networks. To fully utilize the output images from the WCE system, we modify the base network to receive input images with a size of 480 × 480 × 3 without cropping or rescaling.

### 2.4. Loss of Weighted Cross Entropy

Cross-entropy (CE) loss is a common choice for classification tasks. For binary classification [40], CE is defined as

(1) $$\begin{array}{c} \text{CE}\left(p,y\right)=-y\;\log\left(p\right)-\left(1-y\right)\log\left(1-p\right), \end{array}$$

where *y* ∈ {0,  1} denotes the ground-truth label of the sample and *p* ∈ [0,  1] is the estimated probability of a sample belonging to the class with label 1. Mathematically, the minimization process of CE is to enlarge the probabilities of samples with label = 1 and suppress the probabilities of samples with label = 0.

To deal with possible imbalance between classes, a weighting factor can be applied to different classes, which can be called weighted cross-entropy (wCE) loss [41].

(2) $$\begin{array}{c} \text{wCE}\left(p,y;w\right)=-\frac{w}{w+1}\cdot y\;\log\left(p\right)-\frac{1}{w+1}\cdot \left(1-y\right)\log\left(1-p\right), \end{array}$$

where *w* denotes the weighting factor to balance the loss of different classes. Considering the overall small and variational size distribution of ulcers, as well as possible imbalance in the large dataset, we set wCE as our loss function.

### 2.5. Evaluation Criteria

To evaluate the performance of classification, accuracy (AC), sensitivity (SE), and specificity (SP) are exploited as metrics [6].

(3) $$\begin{array}{c} \text{AC}=\frac{\left(\text{TP}+\text{TN}\right)}{N},\\ \text{SE}=\frac{\text{TP}}{\left(\text{TP}+\text{FN}\right)},\\ \text{SP}=\frac{\text{TN}}{\left(\text{TN}+\text{FP}\right)}. \end{array}$$

Here, *N* is the total number of test images and TP, FP, TN, and FN are the number of correctly classified images containing ulcers, the number of normal images falsely classified as ulcer frames, the number of correctly classified images without ulcers, and the number of images with ulcers falsely classified as normal images, respectively.

AC gives an overall assessment of the performance of the model, SE denotes the model's ability to detect ulcer images, and SP denotes its ability to distinguish normal images. Ideally, we expect both high SE and SP, although some trade-offs between these metrics exist. Considering that further manual inspection by the doctor of ulcer images detected by computer-aided systems is compulsory, SE should be as high as possible with no negative impact on overall AC.

**Figure 6:**
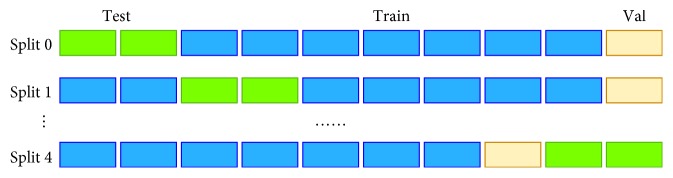
Illustration of cross-validation (green: test dataset; blue: train dataset; yellow: validation dataset).

We use a 5-fold cross-validation strategy at the case level to evaluate the performances of different architectures; this strategy splits the total number of cases evenly into five subsets. Here, one subset is used for testing, and the four other subsets are used for training and validation.  Figure 6 illustrates the cross-validation operation. In the present study, the volumes of train, validation, and test are about 70%, 10%, and 20%, respectively. Normal or ulcer frames are then extracted from each case to form the training/validation/testing dataset. We perform case-level splitting because adjacent frames in the same case are likely to share similar details. We do not conduct frame-level cross-validation splitting to avoid overfitting. The validation dataset is used to select the best model in each training process, i.e., the model with the best validation accuracy during the training iteration is saved as the final model.

## 3. Results

In this section, the implementation process of the proposed method is introduced, and its performance is evaluated by comparison with several other related methods, including state-of-the-art CNN methods and some representative WCE recognition methods based on conventional machine learning techniques.

### 3.1. Network Architectures and Training Configurations

The proposed HAnet connects hyper features to the final feature vector with the aim of enhancing the recognition of ulcers of different sizes. The HAnet models are distinguished by their architecture and training settings, which include three architectures and three training configurations in total. We illustrate these different architectures and configurations in Table 2.

**Figure 7:**
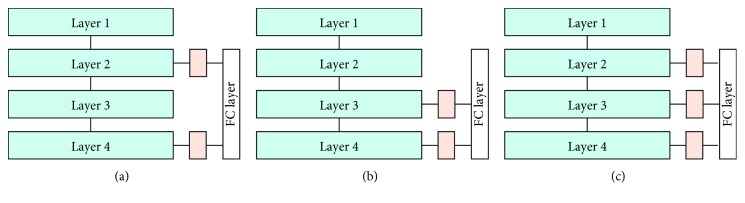
Illustrations of HAnet architectures. (a) hyper(l2), (b) hyper(l3), (c) hyper(l23).

Three different architectures can be obtained when hyper features from layers 2 and 3 are used for decision in combination with features from layer 4 of our ResNet backbone: in Figure 7(a), hyper(l2), which fuses the hyper features from layer 2 with the deep features of layer 4, in Figure 7(b), hyper(l3), which fuses features from layers 3 and 4, and in Figure 7(c), hyper(l23), which fuses features from layers 2, 3, and 4 to form the third HAnet.  Figure 7 provides comprehensive diagrams of these different HAnet architectures.

**Figure 8:**
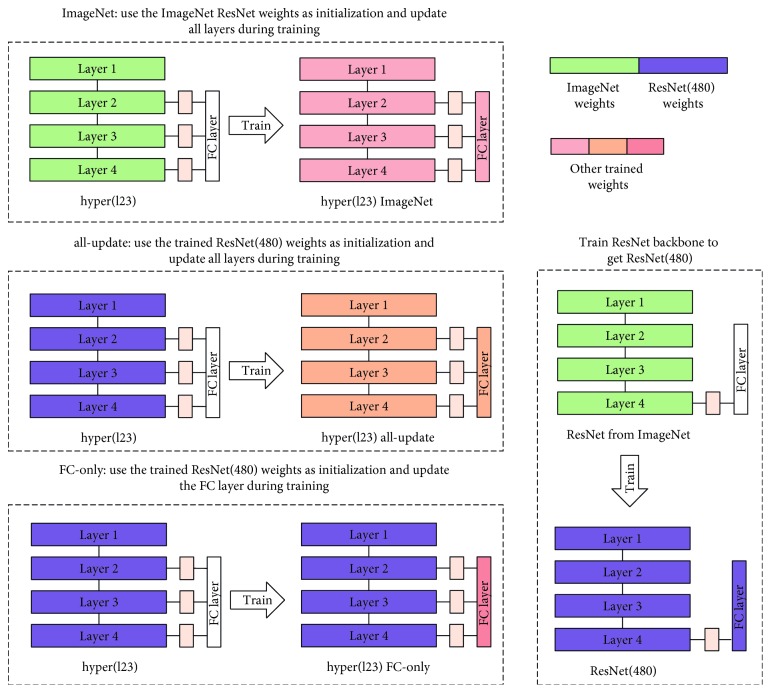
Illustration of three different training configurations, including ImageNet, all-update, and FC-only. Different colors are used to represent different weights. Green denotes pretrained weights from ImageNet, and purple denotes the weights of ResNet(480) that have been fine-tuned on the WCE dataset. Several other colors denote other trained weights.

Each HAnet can be trained with three configurations.  Figure 8 illustrates these configurations.

#### 3.1.1. ImageNet

The whole HAnet is trained using pretrained ResNet weights from ImageNet from initialization (denoted as ImageNet in Table 2). The total training process lasts for 40 epochs, and the batch size is fixed to 16 samples. The learning rate was initialized to be 10^−3^ and decayed by a factor of 10 at each period of 20 epochs. The parameter of momentum is set to 0.9. Experimental results show that 40 epochs are adequate for training to converge. Weighted cross-entropy loss is used as the optimization criterion. The best model is selected based on validation results.

#### 3.1.2. All-Update

ResNet(480) is first fine-tuned on our dataset using pretrained ResNet weights from ImageNet for initialization. The training settings are identical to those in (1). Convergence is achieved during training, and the best model is selected based on validation results. We then train the whole HAnet using the fine-tuned ResNet (480) models for initialization and update all weights in HAnet (denoted as all-update in Table 2). Training lasts for 40 epochs. The learning rate is set to 10^−4^, momentum is set to 0.9, and the best model is selected based on validation results.

#### 3.1.3. FC-Only

The weights of the fine-tuned ResNet(480) model are used, and only the last fully connected (FC) layer is updated in HAnet (denoted as FC-only in Table 2). The best model is selected based on validation results. Training lasts for 10 epochs, the learning rate is set to 10^−4^, and momentum is set to 0.9.

For example, the first HAnet in Table 2, hyper(l2) FC-only, refers to the architecture fusing the features from layer 2 and the final layer 4; it uses ResNet (480) weights as the feature extractor and only the final FC layer is updated during HAnet training.

To achieve better generalizability, data augmentation was applied online in the training procedure as suggested in [7]. The images are randomly rotated between 0° and 90° and flipped with 50% possibility. Our network is implemented using PyTorch.

The experiments are conducted on an Intel Xeon machine (Gold 6130 CPU@2.10 GHz) with Nvidia Quadro GP100 graphics cards. Detailed results are presented in Sections 3.2–3.4.

### 3.2. Refinement of the Weighting Factor for Weighted Cross-Entropy

**Figure 9:**
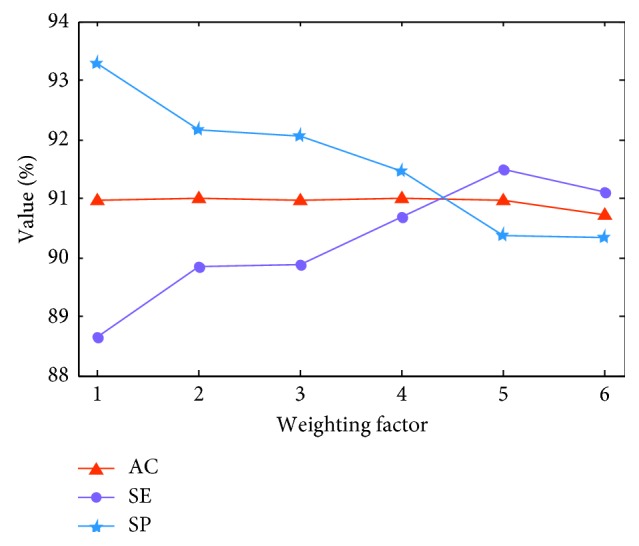
AC, SE, and SP evolution against the wCE weighting factor. Red, purple, and blue curves denote the results of AC, SE, and SP, respectively. The horizontal axis is the weighting factor, and the vertical axis is the value of AC, SE, and SP.

To demonstrate the impact of different weighting factors, i.e., *w* in equation (2), we examine the cross-validation results of model recognition accuracy with different weighting factors. The AC, SE, and SP curves are shown in Figure 9.

AC varies with changes in weighting factor. In general, SE improves while SP is degraded as the weighting factor increases. Detailed AC, SE, and SP values are listed in Table 3. ResNet-18(480) refers to experiments on a ResNet-18 network with a WCE full-resolution image input of 480 × 480 × 3. A possible explanation for the observed effect of the weighting factor is that the ulcer dataset contains many consecutive frames of the same ulcer, and these frames may share marked similarities. Thus, while the frame numbers of the ulcer and normal dataset are comparable, the information contained by each dataset remains unbalanced. The weighting factor corrects or compensates for this imbalance.

In the following experiments, 4.0 is used as the weighting factor as it outperforms other choices and simultaneously achieves good balance between SE and SP.

### 3.3. Selection of Hyper Architectures

We tested 10 models in total, as listed in Table 4, including a ResNet-18(480) model and nine HAnet models based on ResNet-18(480). The resolution of input images is 480 × 480 × 3 for all models.

According to the results in Table 4, the FC-only and all-update hyper models consistently outperform the ResNet-18(480) model in terms of the AC criterion, which demonstrates the effectiveness of HAnet architectures. Moreover, FC-only models generally perform better than all-update models, thus implying that ResNet-18(480) extracts features well and that further updates may corrupt these features.

The hyper ImageNet models, including hyper(l2) ImageNet, hyper(l3) ImageNet, and hyper(l23) ImageNet, seem to give weak performance. Hyper ImageNet models and the other hyper models share the same architectures. The difference between these types of models is that the hyper ImageNet models are trained with the pretrained ImageNet ResNet-18 weights while the other models use ResNet-18(480) weights that have been fine-tuned on the WCE dataset. This finding reveals that a straightforward base net such as ResNet-18(480) shows great power in extracting features. The complicated connections of HAnet may prohibit the network from reaching good convergence points.

To fully utilize the advantages of hyper architectures, we recommend a two-stage training process: (1) Train a ResNet-18(480) model based on the ImageNet-pretrained weights and then (2) use the fine-tuned ResNet-18(480) model as a backbone feature extractor to train the hyper models. We denote the best model in all hyper architectures as HAnet-18(480), i.e., a hyper(l23) FC-only model.

**Figure 10:**
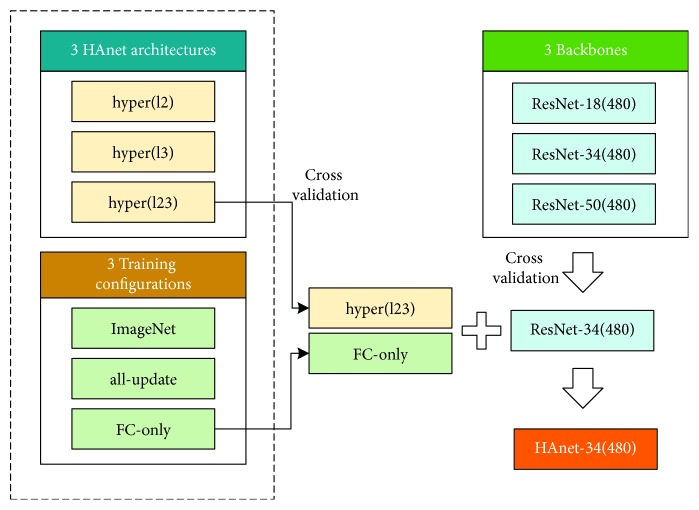
Progression of HAnet-34(480).

Additionally, former exploration is based on ResNet-18, and results indicate that a hyper(l23) FC-only architecture based on the ResNet backbone feature extractor fine-tuned by WCE images may be expected to improve the recognition capability of lesions in WCE videos. To optimize our network, we examine the performance of various ResNet series members to determine an appropriate backbone. The corresponding results are listed in Table 5; ResNet-34(480) has better performance than ResNet-18(480) and ResNet-50(480). Thus, we take ResNet-34(480) as our backbone to train HAnet-34(480). The training settings are described in Section 3.1.  Figure 10 gives the overall progression of HAnet-34(480).

### 3.4. Comparison with Other Methods

To evaluate the performance of HAnet, we compared the proposed method with several other methods, including several off-the-shelf CNN models [26–28] and two representative handcrafted-feature based methods for WCE recognition [3, 42]. The off-the-shelf CNN models, including VGG [28], DenseNet [26], and Inception-ResNet-v2 [27], are trained to converge with the same settings as ResNet-34(480), and the best model is selected based on the validation results. For handcrafted-feature based methods, grid searches to optimize hyper parameters are carried out.

**Figure 11:**
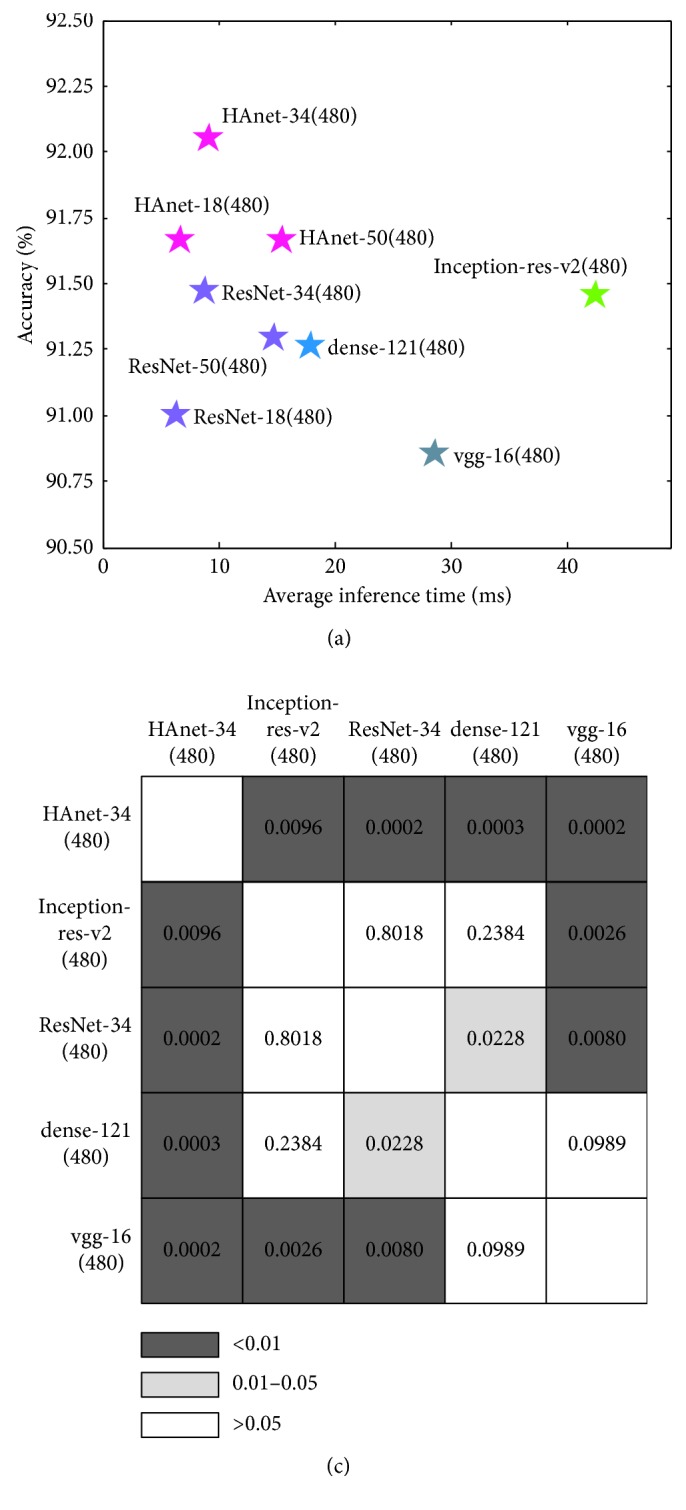
Comparison of different models. (a) Accuracy and inference time comparison. The horizontal and vertical axes denote the inference time and test accuracy, respectively. (b) Statistical test results of paired *T*-Test. Number in each grid cell denotes the *p* value of the two models in the corresponding row and column.

We performed repeated 2 × 5-fold cross-validation to provide sufficient measurements for statistical tests.  Table 6 compares the detailed results of HAnet-34(480) with those of other methods. On average, HAnet-34(480) performs better in terms of AC, SE, and SP than the other methods.  Figure 11(a) gives the location of each model, considering its inference time and accuracy.  Figure 11(b) is the statistical results of paired *T*-Test.

Among the models tested, HAnet-34(480) yields the best performance with good efficiency and accuracy. Additionally, the statistical test results demonstrate the improvement of our HAnet-34 is statistically significant. Number in each grid cell denotes the *p* value of the two models in the corresponding row and column. We can see that the improvement of HAnet-34(480) is statistically significant at the 0.01 level compared with other methods.

Table 7 gives more evaluation results based on several criteria, including precision (PRE), recall (RECALL), F1 and F2 scores [33], and ROC-AUC [6]. HAnet-34 outperforms all other models based on these criteria.

## 4. Discussion

In this section, the recognition capability of the proposed method for small lesions is demonstrated and discussed. Recognition results are also visualized via the class activation map (CAM) method [43], which indicates the localization potential of CNN networks for clinical diagnosis.

### 4.1. Enhanced Recognition of Small Lesions by HAnet

To analyze the recognition capacity of the proposed model, the sensitivities of ulcers of different sizes are studied, and the results of ResNet-34(480) and the best hyper model, HAnet-34(480), are listed in Table 8.

Based on the results of each row in Table 8, most of the errors noticeably occur in the small size range for both models. In general, the larger the ulcer, the easier its recognition. In the vertical comparison, the ulcer recognition of HAnet-34(480) outperforms that of ResNet-34(480) at all size ranges including small lesions.

### 4.2. Visualization of Recognition Results

**Figure 12:**
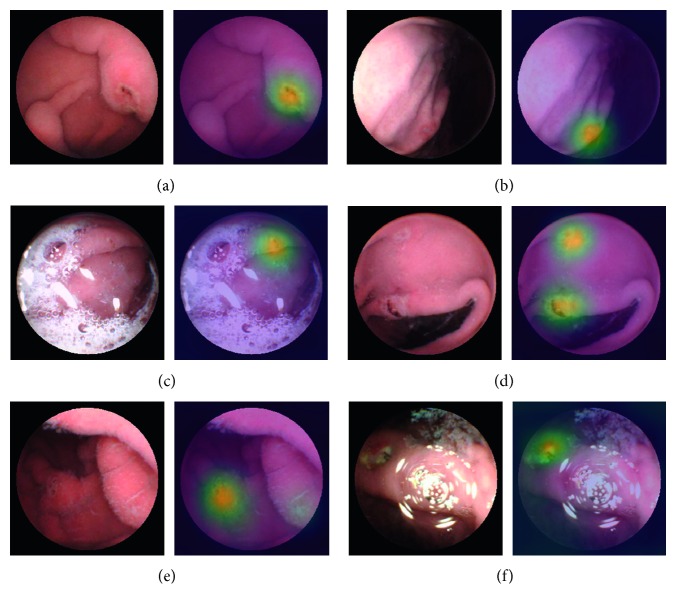
Visualization network results of some representative ulcers with CAM. A total of six groups of representative frames are obtained. For each group, the left image reflects the original frame, while the right image shows the result of CAM. (a) Typical ulcer, (b) ulcer on the edge, (c) ulcer in a turbid background with bubbles, (d) multiple ulcers in one frame, (e) ulcer in a shadow, and (f) ulcer recognition in the frame with a flashlight.

To better understand our network, we use a CAM [43] generated from GAP to visualize the behavior of HAnet by highlighting the relatively important parts of an image and providing object location information. CAM is the weighted linear sum of the activation map in the last convolutional layer. The image regions most relevant to a particular category can be simply obtained by upsampling the CAM. Using CAM, we can verify what indeed has been learned by the network. Six cases of representative results are displayed in Figure 12. For each pair of images, the left image shows the original frame, while the right image shows the CAM result.

These results displayed in Figure 12 demonstrate the potential use of HAnet for locating ulcers and easing the work of clinical physicians.

## 5. Conclusion

In this work, we proposed a CNN architecture for ulcer detection that uses a state-of-the-art CNN architecture (ResNet-34) as the feature extractor and fuses hyper and deep features to enhance the recognition of ulcers of various sizes. A large ulcer dataset containing WCE videos from 1,416 patients was used for this study. The proposed network was extensively evaluated and compared with other methods using overall AC, SE, SP, F1, F2, and ROC-AUC as metrics.

Experimental results demonstrate that the proposed architecture outperforms off-the-shelf CNN architectures, especially for the recognition of small ulcers. Visualization with CAM further demonstrates the potential of the proposed architecture to locate a suspicious area accurately in a WCE image. Taken together, the results suggest a potential method for the automatic diagnosis of ulcers from WCE videos.

Additionally, we conducted experiments to investigate the effect of number of cases. We used split 0 datasets in the cross-validation experiment, 990 cases for training, 142 cases for validation, and 283 cases for testing. We constructed different training datasets from the 990 cases while fixed the validation and test dataset. Firstly, we did experiments on using different number of cases for training. We randomly selected 659 cases, 423 cases, and 283 cases from 990 cases. Then, we did another experiment using similar number of frames as last experiment that distributed in all 990 cases. Results demonstrate that when similar number of frames are used for training, test accuracies using training datasets with more cases are better. This should be attributed to richer diversity introduced by more cases. We may recommend to use as many cases as possible to train the model.

While the performance of HAnet is very encouraging, improving its SE and SP further is necessary. For example, the fusion strategy in the proposed architecture involves concatenation of features from shallow layers after GAP. Semantic information in hyper features may not be as strong as that in deep features, i.e., false-activated neural units due to the relative limited receptive field in the shallow layers may add unnecessary noise to the concatenated feature vector when GAP is utilized. An attention mechanism [44] that can focus on the suspicious area may help address this issue. Temporal information from adjacent frames could also be used to provide external guidance during recognition of the current frame. In future work, we will explore more techniques in network design to improve ulcer recognition.

## Figures and Tables

**Table 1:** Representative studies and datasets of WCE videos in the literature.

Experiment	Cases	Detail
Li and Meng [5]	10 patients' videos	10 patients' videos, 200 images
Li and Meng [16]	10 patients' videos	10 patients' videos (five for bleeding and the other five for ulcer)
Li et al. [17]	—	80 representative small intestine ulcer WCE images and 80 normal images
Karargyris and Bourbakis [9]	—	A WCE video containing 10 frames with polyps and 40 normal frames and extra 20 frames with ulcer
Li and Meng [8]	10 patients' videos	10 patients' videos, 600 representative polyp images and 600 normal images from data; 60 normal images and 60 polyp images from each patient's video segments
Yu et al. [10]	60 patients' videos	60 patients' videos, 344 endoscopic images for training; another 120 ulcer images and 120 normal images for testing
Fu et al. [12]	20 patients' videos	20 patients' videos, 5000 WCE images consisting of 1000 bleeding frames and 4000 nonbleeding frames
Yeh et al. [11]	—	607 images containing 220, 159, and 228 images of bleeding, ulcers, and nonbleeding/ulcers, respectively
Yuan et al. [3]	10 patients' videos	10 patients' videos, 2400 WCE images that consist of 400 bleeding frames and 2000 normal frames
Yuan and Meng [7]	35 patients' videos	35 patients' videos, 3000 normal WCE images (1000 bubbles, 1000 TIs, and 1000 CIs) and 1000 polyp images
He et al. [6]	11 patients' videos	11 patients' videos, 440K WCE images
Aoki et al. [18]	180 patients' videos	115 patients' videos, 5360 images of small-bowel erosions and ulcerations for training; 65 patients' videos, 10,440 independent images for validation
Ours	1,416 patients' videos	1,416 patients' videos with 24,839 representative ulcer frames

**Table 2:** Illustration of different architectures and configurations.

Model	Features			Network initialization weights	Training
	Layer 2	Layer 3	Layer 4		
ResNet(480)			✓	ImageNet	Train on WCE dataset

hyper(l2) FC-only	✓		✓	ResNet(480)	Update FC-layer only
hyper(l3) FC-only		✓	✓		
hyper(l23) FC-only	✓	✓	✓		

hyper(l2) all-update	✓		✓	ResNet(480)	Update all layers
hyper(l3) all-update		✓	✓		
hyper(l23) all-update	✓	✓	✓		

hyper(l2) ImageNet	✓		✓	ImageNet	Update all layers
hyper(l3) ImageNet		✓	✓		
hyper(l23) ImageNet	✓	✓	✓		

**Table 3:** Cross-validation accuracy of ResNet-18(480) with different weighting factors.

Weighting factor (*w*)	1.0	2.0	3.0	4.0	5.0	6.0
AC (%)	90.95 ± 0.64	91.00 ± 0.49	90.96 ± 0.68	91.00 ± 0.70	90.95 ± 0.83	90.72 ± 0.75
SE (%)	88.65 ± 0.64	89.85 ± 0.47	89.86 ± 1.01	90.67 ± 0.93	91.50 ± 0.76	91.12 ± 1.94
SP (%)	93.27 ± 1.05	92.15 ± 1.22	92.05 ± 1.09	91.45 ± 1.84	90.38 ± 1.26	90.32 ± 1.88

**Table 4:** Performances of different architectures.

Model	Cross validation		
	AC (%)	SE (%)	SP (%)
ResNet-18(480)	91.00 ± 0.70	90.55 ± 0.86	91.45 ± 1.84
hyper(l2) FC-only	91.64 ± 0.79	91.22 ± 1.08	92.05 ± 1.65
hyper(l3) FC-only	91.62 ± 0.65	91.15 ± 0.56	92.07 ± 1.45
**hyper(l23) FC-only**	**91.66** ** ± 0.81**	**91.48** ** ± 0.90**	**91.83** ** ± 1.75**
hyper(l2) all-update	91.39 ± 1.02	91.28 ± 1.04	91.47 ± 1.86
hyper(l3) all-update	91.50 ± 0.81	90.63 ± 1.06	92.33 ± 1.74
hyper(l23) all-update	91.37 ± 0.72	91.33 ± 0.63	91.4 ± 1.42
hyper(l2) ImageNet	90.96 ± 0.90	90.52 ± 1.10	91.38 ± 2.01
hyper(l3) ImageNet	91.04 ± 0.80	90.52 ± 1.34	91.54 ± 1.31
hyper(l23) ImageNet	90.82 ± 0.85	90.26 ± 1.33	91.37 ± 1.48

**Table 5:** Model recognition accuracy with different settings.

	ResNet-18(480)	ResNet-34(480)	ResNet-50(480)
AC (%)	91.00 ± 0.70	**91.50** ± 0.70	91.29 ± 0.91
SE (%)	90.55 ± 0.86	90.74 ± 0.74	89.63 ± 1.83
SP (%)	91.45 ± 1.84	92.25 ± 1.72	92.94 ± 1.82

**Table 6:** Comparison of HAnet with other methods.

	AC (%)	SE (%)	SP (%)
SPM-BoW-SVM [42]	61.38 ± 1.22	51.47 ± 2.65	70.67 ± 2.24
Words-based-color-histogram [3]	80.34 ± 0.29	82.21 ± 0.39	78.44 ± 0.29
vgg-16(480) [28]	90.85 ± 0.98	90.12 ± 1.17	92.02 ± 2.52
dense-121(480) [26]	91.26 ± 0.43	90.47 ± 1.67	92.07 ± 2.04
Inception-ResNet-v2(480) [27]	91.45 ± 0.80	90.81 ± 1.95	92.12 ± 2.71
ResNet-34(480) [25]	91.47 ± 0.52	90.53 ± 1.14	92.41 ± 1.66
HAnet-34(480)	**92.05** ± **0.52**	**91.64** ± **0.95**	**92.42** ± **1.54**

**Table 7:** Evaluation of different criteria.

	PRE	RECALL	F1	F2	ROC-AUC
vgg-16(480)	0.9170	0.9012	0.9087	0.9040	0.9656
dense-121(480)	0.9198	0.9047	0.9118	0.9074	0.9658
Inception-ResNet-v2(480)	0.9208	0.9081	0.9138	0.9102	0.9706
ResNet-34(480)	0.9218	0.9053	0.9133	0.9084	0.9698
HAnet-34(480)	**0.9237**	**0.9164**	**0.9199**	**0.9177**	**0.9726**

**Table 8:** Recognition of ulcer with different sizes.

Model	Ulcer size			
	<1%	1–2.5%	2.5–5%	>5%
ResNet-34(480)	81.44 ± 3.07	91.86 ± 1.40	94.16 ± 1.26	96.51 ± 1.43
HAnet-34(480)	82.37 ± 3.60	92.78 ± 1.33	95.40 ± 0.74	97.11 ± 1.11

**Table 9:** Architectures of ResNet series members.

Layer name	Output size	18-Layer	34-Layer	50-Layer	101-Layer	152-Layer
Conv1	240 × 240	7 × 7, 64, stride 2				

Maxpool	120 × 120	3 × 3, max pool, stride 2				

Layer 1	120 × 120	$\left[\begin{array}{cc} 3\times 3 & 64\\ 3\times 3 & 64 \end{array}\right]\times 2$	$\left[\begin{array}{cc} 3\times 3 & 64\\ 3\times 3 & 64 \end{array}\right]\times 3$	$\left[\begin{array}{cc} 1\times 1 & 64\\ 3\times 3 & 64\\ 1\times 1 & 256 \end{array}\right]\times 4$	$\left[\begin{array}{cc} 1\times 1 & 64\\ 3\times 3 & 64\\ 1\times 1 & 256 \end{array}\right]\times 3$	$\left[\begin{array}{cc} 1\times 1 & 64\\ 3\times 3 & 64\\ 1\times 1 & 256 \end{array}\right]\times 3$
Layer 2	60 × 60	$\left[\begin{array}{cc} 3\times 3 & 128\\ 3\times 3 & 128 \end{array}\right]\times 2$	$\left[\begin{array}{cc} 3\times 3 & 128\\ 3\times 3 & 128 \end{array}\right]\times 4$	$\left[\begin{array}{cc} 1\times 1 & 128\\ 3\times 3 & 128\\ 1\times 1 & 512 \end{array}\right]\times 4$	$\left[\begin{array}{cc} 1\times 1 & 128\\ 3\times 3 & 128\\ 1\times 1 & 512 \end{array}\right]\times 4$	$\left[\begin{array}{cc} 1\times 1 & 128\\ 3\times 3 & 128\\ 1\times 1 & 512 \end{array}\right]\times 8$
Layer 3	30 × 30	$\left[\begin{array}{cc} 3\times 3 & 256\\ 3\times 3 & 256 \end{array}\right]\times 2$	$\left[\begin{array}{cc} 3\times 3 & 128\\ 3\times 3 & 128 \end{array}\right]\times 6$	$\left[\begin{array}{cc} 1\times 1 & 256\\ 3\times 3 & 256\\ 1\times 1 & 1024 \end{array}\right]\times 6$	$\left[\begin{array}{cc} 1\times 1 & 256\\ 3\times 3 & 256\\ 1\times 1 & 1024 \end{array}\right]\times 23$	$\left[\begin{array}{cc} 1\times 1 & 256\\ 3\times 3 & 256\\ 1\times 1 & 1024 \end{array}\right]\times 36$
Layer 4	15 × 15	$\left[\begin{array}{cc} 3\times 3 & 512\\ 3\times 3 & 512 \end{array}\right]\times 2$	$\left[\begin{array}{cc} 3\times 3 & 512\\ 3\times 3 & 512 \end{array}\right]\times 3$	$\left[\begin{array}{cc} 1\times 1 & 512\\ 3\times 3 & 512\\ 1\times 1 & 2048 \end{array}\right]\times 3$	$\left[\begin{array}{cc} 1\times 1 & 512\\ 3\times 3 & 512\\ 1\times 1 & 2048 \end{array}\right]\times 3$	$\left[\begin{array}{cc} 1\times 1 & 512\\ 3\times 3 & 512\\ 1\times 1 & 2048 \end{array}\right]\times 3$

Avgpool and fc	1 × 1	Global average pool, 2-d fc				

## Data Availability

The WCE datasets used to support the findings of this study are available from the corresponding author upon request.

## Appendix

The architectures of members of the ResNet series, including ResNet-18, ResNet-34, ResNet-50, ResNet-101, and ResNet-152, are illustrated in Table 9. These members share a similar pipeline consisting of seven components (i.e., conv1, maxpool, layer 1, layer 2, layer 3, layer 4, and avgpool + fc). Each layer in layers 1–4 has different subcomponents denoted as [ ] × *n*, where [ ] represents a specific basic module consisting of 2-3 convolutional layers and *n* is the number of times the basic module is repeated. Each row in [ ] describes the convolution layer setting, e.g., [(3 × 3)64] means a convolution layer with a kernel size and channel of 3 × 3 and 64, respectively.
